# Constructing Sulfur Vacancy-Rich NiCo_2_S_4_@MoS_2_ Core@shell Heterostructure via Interface Engineering for Enhanced HER Electrocatalysis

**DOI:** 10.3390/nano15141061

**Published:** 2025-07-09

**Authors:** Ziteng Song, Yuan Liu, Peng Yin, Jie Dai, Yingying Xu, Rongming Wang, Sibin Duan

**Affiliations:** Beijing Key Laboratory for Magneto-Photoelectrical Composite and Interface Science, The State Key Laboratory for Advanced Metals and Materials, School of Mathematics and Physics, University of Science and Technology Beijing, Beijing 100083, China; m202210768@xs.ustb.edu.cn (Z.S.); d202420044@xs.ustb.edu.cn (Y.L.); pengyin@ruc.edu.cn (P.Y.); jiedai@xs.ustb.edu.cn (J.D.); xuyingying@ustb.edu.cn (Y.X.)

**Keywords:** heterostructure, core@shell nanostructure, sulfur vacancies, interface engineering

## Abstract

The rational design of heterointerfaces with optimized charge dynamics and defect engineering remains pivotal for developing advanced non-noble metal-based electrocatalysts for water splitting. A comparative study of NiCo_2_S_4_–MoS_2_ heterostructures was conducted to elucidate the impact of interfacial architecture and defect engineering on hydrogen evolution reaction (HER) performance. A core@shell NiCo_2_S_4_@MoS_2_ heterostructure was synthesized via a facile hydrothermal growth method, inducing lattice distortion and strong interfacial coupling, while supported NiCo_2_S_4_/MoS_2_ heterostructures were prepared by ultrasonic-assisted deposition. A detailed structural and spectroscopic characterization and theoretical calculation demonstrated that the core@shell configuration promotes charge redistribution across the NiCo_2_S_4_–MoS_2_ interface and generates abundant sulfur vacancies, thereby increasing the density of electroactive sites. Electrochemical measurements reveal that NiCo_2_S_4_@MoS_2_ markedly outperforms the supported heterostructure, single-component NiCo_2_S_4_, and MoS_2_ when serving as the HER catalyst in acid solution. These findings establish a dual-optimization strategy—combining interfacial design with vacancy modulation—that provides a generalizable paradigm for the deliberate design of high-efficiency non-noble metal-based electrocatalysts for water splitting reactions.

## 1. Introduction

To solve energy shortages and environmental degradation, advancing renewable energy and minimizing the dependence on fossil fuels have emerged as urgent global imperatives [1,2]. Hydrogen energy, being a clean and renewable energy source, has numerous advantages. Hydrogen combustion yields water and does not generate greenhouse gases. It possesses a high energy density, making it appropriate for extensive storage and transportation [3,4,5]. Among the various hydrogen production methods, water splitting via electrolysis has become a major focus in recent research due to its simple production process and environmentally friendly nature [6,7,8]. However, the high overpotentials of the hydrogen evolution reaction (HER) and oxygen evolution reaction (OER) significantly limit the overall efficiency of water electrolysis. For widely used non-noble transition metal-based catalysts (including Ni-, Co-, and Fe-based compounds), this is essentially related to insufficient charge kinetics and a limited number of defect-mediated active sites [9,10,11]. Developing highly efficient and cost-effective electrocatalysts to improve water splitting performance has thus become a critical task.

Conventional Pt-based catalysts (e.g., Pt/C) exhibit excellent HER activity but suffer from high costs and resource scarcity. In contrast, non-noble transition metal-based catalysts (including Ni-, Co-, and Fe-based compounds) are more cost-effective and abundant in resources [12,13,14]. Among them, Co/Ni sulfides have emerged as promising HER catalysts due to their multiple valence states and tunable electronic structures [15,16,17,18,19]. Ding et al. demonstrated that NiS experienced a phase transformation to a Ni_3_S_2_/NiO heterointerface during an in situ alkaline HER catalytic reaction, as revealed by near-ambient pressure X-ray photoelectron spectroscopy (NAP-XPS) and operando X-ray absorption spectroscopy. The Ni sites facilitate water dissociation and OH* adsorption, while adjacent S sites optimize H* binding energy, achieving a relatively low overpotential of 95 mV at 10 mA cm^−2^ [18]. However, the inherent conductivity of single-component transition metal sulfides is comparatively low, restricting charge transfer efficiency and resulting in sluggish reaction kinetics. Furthermore, its capacity for the adsorption and dissociation of water molecules is relatively weak, leading to a limited overall reaction rate [20,21,22]. For instance, the high energy barrier for water dissociation hinders the electrochemical performance of single-component Ni_3_S_2_ [23]. In contrast, bimetallic sulfides incorporating two metal elements exhibit enhanced electrocatalytic properties resulting from the lower activation barriers for inter-cation electron transfer, the presence of mixed valence states, and a higher density of active sites [24,25]. They also demonstrate enhanced electrical conductivity and improved electrochemical structural stability, making them promising candidates for next-generation water splitting electrocatalysts [26,27,28,29,30]. Ding et al. synthesized NiCo@NiS nanoparticles anchored on S-doped CNTs, where the Ni–Co dual-metal synergy optimizes interfacial charge redistribution and stabilizes sulfur-induced active sites. This bimetallic configuration achieves an HER overpotential of −1.16 V vs. Ag/AgCl at 10 mA cm^−2^, outperforming monometallic counterparts, as Co incorporation mitigates Ni’s strong H* adsorption. At the same time, S doping enhances CNT conductivity and site accessibility [26].

Meanwhile, molybdenum disulfide (MoS_2_), a representative 2D layered dichalcogenide, is considered a viable substitute for Pt-based HER catalysts owing to its favorable hydrogen adsorption free energy at edge sites, which is comparable to that of Pt [31,32]. However, the effective utilization of pristine MoS_2_ is constrained by several intrinsic limitations, including its catalytically inert basal planes, unfavorable hydrogen adsorption free energy (ΔG_H*_), and limited intrinsic conductivity [33]. Critically, the HER efficiency fundamentally depends on the binding strength of the hydrogen intermediate (H*) to the active sites, ideally characterized by a ΔG_H*_ close to zero [34,35]. To activate the inert basal planes and optimize ΔG_H*_, defect engineering, particularly the introduction of sulfur vacancies (S_V_s), has emerged as a powerful strategy [20]. S_V_s expose coordinatively unsaturated Mo atoms, which can serve as highly active sites. More importantly, S_V_s act as n-type dopants, profoundly modifying the local electronic structure of MoS_2_, introducing gap states and shifting the d-band center [36]. These electronic modifications are pivotal for tuning the adsorption strength of H* towards the optimal value. Simultaneously, S_V_s significantly enhance the electrical conductivity, facilitating charge transfer to active sites [37,38]. Xu et al. engineered monolayer MoS_2_ with Frenkel defects, where displaced Mo atoms create dual vacancies and interstitial sites, inducing localized charge redistribution. These interstitial Mo atoms optimize H* adsorption, achieving an HER overpotential of 164 mV at 10 mA cm^−2^, which is 54% lower than pristine MoS_2_ and 22% below Pt-doped counterparts [39]. Besides the defect engineering, constructing heterostructures with conductive materials offers another effective method to enhance MoS_2_-based HER electrocatalysts [20]. The formation of a heterojunction induces interfacial charge redistribution due to differences in work function, leading to band bending and often creating an interfacial electric field [40,41]. This interfacial electronic structure reconstruction can significantly modulate the electronic states and catalytic properties of active sites at the interface, potentially optimizing their ΔG_H*_ [42].

Based on these points, NiCo_2_S_4_-MoS_2_ heterostructures were designed via a two-step wet chemical approach, yielding both a MoS_2_-coated NiCo_2_S_4_@MoS_2_ core@shell architecture and a physically supported NiCo_2_S_4_/MoS_2_ heterostructure. Structural characterization confirmed intimate interfacial contact in the core@shell structure and a high density of S_V_s at the NiCo_2_S_4_–MoS_2_ interfaces and surfaces. Electrochemical evaluation revealed that the NiCo_2_S_4_@MoS_2_ core@shell catalyst possesses superior HER activity, resulting from efficient interfacial electron transfer and an abundance of S_V_s that increase the electroactive site density. This work demonstrates that precise interfacial engineering coupled with defect modulation provides a reliable foundation for the development of high-efficiency non-noble metal electrocatalysts for water splitting.

## 2. Materials and Methods

**Materials:**

The nickel acetylacetonate (Ni(acac)_2_), cobalt acetylacetonate (Co(acac)_3_), oleylamine (OAm), thiourea, sodium molybdate dihydrate (Na_2_MoO_4_·2H_2_O), and MoS_2_ nanosheets were purchased from Shanghai Macklin Biochemical Co., Ltd. (Shanghai, China). Nafion (5 wt%) solution was purchased from Shanghai Hesen Electric Co., Ltd. (Shanghai, China). All reagents were directly used without further purification.

**Synthesis of NiCo_2_S_4_ nanoparticles:**

In a typical synthesis, Ni(acac)_2_ (0.05 g) and Co(acac)_3_ (0.10 g) were dissolved in OAm (16 mL) and transferred to a three-neck flask. The mixture was further heated up to 210 °C while maintaining continuous stirring to produce a homogenous solution. Subsequently, thiourea (0.123 g) was added to the solution, and the temperature was elevated to 240 °C, with electromagnetic stirring maintained for 150 min. High-purity argon gas was consistently provided as a protective environment during the reaction. After the reaction, the solution was allowed to cool to ambient temperature and was collected using centrifugation. The resultant precipitate was thoroughly washed multiple times with acetone and chloroform, followed by vacuum drying at 60 °C for 8 h. The dehydrated product was finally ground into a black powder.

**Synthesis of NiCo_2_S_4_@MoS_2_ core@shell heterostructure:**

The NiCo_2_S_4_@MoS_2_ core@shell heterostructure was synthesized by a hydrothermal method. Initially, 25.0 mg of dehydrated NiCo_2_S_4_ nanoparticles was dispersed in 5.0 mL of anhydrous ethanol using ultrasonication for 20 min, during which 20 mL of deionized water was gradually added dropwise. Subsequently, 27.0 mg of Na_2_MoO_4_·2H_2_O and 55.4 mg of thiourea were added as the Mo and S sources. The mixture was subjected to further ultrasonication for 30 min to achieve uniform dispersion. The produced solution was then transferred to an autoclave and heated at 190 °C for 18 h. Upon natural cooling to ambient temperature, the product was obtained using centrifugation, thoroughly washed with ethanol and deionized water, and then vacuum-dried at 60 °C for 8 h. The dehydrated product was finally ground into a black powder.

**Synthesis of NiCo_2_S_4_/MoS_2_ supported heterostructure:**

The NiCo_2_S_4_/MoS_2_ supported heterostructure was generated using an ultrasonic-assisted deposition method. Firstly, 10.0 mg of dehydrated NiCo_2_S_4_ nanoparticles was dispersed in 5.0 mL of chloroform, while 25.0 mg of MoS_2_ nanosheets was dispersed in 20 mL of ethanol. Both solutions underwent ultrasonication for 30 min to achieve optimal dispersion before mixing. The resulting combination was subjected to ultrasonication for 2 h, during which high-purity argon gas was continuously supplied to maintain the fluidity of the solution. Subsequently, the product was acquired through centrifugation, thoroughly washed with anhydrous ethanol, and vacuum-dried at 60 °C for 8 h. The dehydrated product was finally ground into a powder.

**Sample Characterization:**

The crystal structures of the samples were examined utilizing a powder X-ray diffractometer (XRD; Malvern Panalytical X’Pert Powder diffractometer, Almelo, The Nederlands; Cu Kα radiation, *λ* = 0.15406 nm). The morphologies and microstructures were analyzed using transmission electron microscopy (TEM; JEOL, JEM-2200FS, Tokyo, Japan) at an acceleration voltage of 200 kV. The elemental compositions and distributions were characterized using energy-dispersive X-ray spectroscopy (EDS; Oxford Instruments, X-Max80T, Oxford, UK) in conjunction with TEM. The specimens used for the TEM analysis were prepared by spreading the material onto a copper grid coated with a thin layer of holey carbon. The electronic structures were analyzed using XPS (PHI 5000 Versa Probe III spectrometer, Kanagawa, Japan; Al Kα X-ray source, 1486.6 eV). The precise NiCo_2_S_4_:MoS_2_ mass ratios of the prepared samples were determined using inductively coupled plasma mass spectrometry (ICP-MS; Agilent ICPOES730, Santa Clara, CA, USA).

**Electrocatalytic Performance Evaluation:**

The electrocatalytic performances of the samples were assessed utilizing a standard three-electrode electrochemical workstation (Shanghai Chenhua Instrument Co., Ltd., CHI 760E, Shanghai, China).

The preparation of the working electrode involved mixing 5.0 mg of the catalyst with 5.0 mg of carbon black in 1.0 mL of anhydrous ethanol, after which 100 μL of a 5 wt% Nafion solution was added. The resulting suspension underwent magnetic stirring for 15 min to achieve a homogeneous ink. Following this, 44 μL of the ink was carefully drop-cast onto a 1 cm^2^ piece of carbon fiber paper and allowed to dry at ambient temperature. The final catalyst loading on the electrode was around 0.2 mg cm^−2^.

Electrochemical testing was conducted using a standard three-electrode configuration, with a carbon rod as the counter electrode and a saturated Ag/AgCl electrode as the reference. The electrocatalytic activity was examined using linear sweep voltammetry (LSV) at a scan rate of 5 mV s^−1^ with 90% *iR* compensation. The measured potentials were converted to the reversible hydrogen electrode (RHE) scale using the following equation:

*E*_vs. RHE_ = *E*_vs. Ag/AgCl_ + 0.059 × pH + 0.224.

The electrochemical impedance spectroscopy (EIS) measurements were performed utilizing the frequency response analyzer in potentiostatic mode. During this process, the working electrode was subjected to an AC voltage amplitude of 2 mV, and data were collected over a frequency range from 0.1 Hz to 100 kHz. The double-layer capacitance (*C*_dl_) served as a parameter for estimating the electrochemical active surface area (ECSA). The data is obtained from the cyclic voltammetry (CV) curves recorded at scan rates of 20, 40, 60, 80, and 100 mV s^−1^. The catalyst’s long-term operational stability was assessed through chronoamperometric measurements (*i*-*t* curves) performed at a constant potential of −0.507 V.

**Computational details:**

All density functional theory (DFT) calculations utilized the Vienna Ab initio Simulation Package (VASP), employing the Perdew–Burke–Ernzerhof (PBE) functional and the projector-augmented wave (PAW) method to characterize the Gibbs free energy of the HER [43,44]. The wave functions were utilized within a plane wave basis set featuring an energy cutoff of 520 eV. The convergence requirement for the self-consistency process was established at 10^−5^ eV between two electronic iterations, with residual forces in each direction remaining below 0.01 eV Å^−1^. During structural relaxations, the lowest three atomic layers of NiCo_2_S_4_ were constrained, while all other atoms were allowed to relax. VASPKIT was employed to examine the computed outcomes of VASP [45]. The optimized monolayer 2H-MoS_2_ with the lowest energy was enlarged into a 4 × 4 supercell and positioned on the NiCo_2_S_4_ (111) surface to create the NiCo_2_S_4_–MoS_2_ heterostructure. A sulfur vacancy was created on the lower surface of MoS_2_ to produce the NiCo_2_S_4_–MoS_2−*x*_ structures. The Brillouin zone was sampled using a *k*-point mesh of 3 × 3 × 1. All slab models were separated by a vacuum of 20 Å to provide decoupling between adjacent slabs.

The Gibbs free energy shift for each elemental stage of the HER was determined using the computational hydrogen electrode (CHE) model presented by Nørskov et al., expressed as [46]:

∆*G* = ∆*E* + ∆*E*_ZPE_ − *T*∆*S*,

where ∆*E*, ∆*E*_ZPE_, and ∆*S* are the energy obtained directly from DFT calculations, change in zero-point energy, and change in entropy terms, respectively, with temperature *T* = 298.15 K.

## 3. Results and Discussion

**Figure 1:**
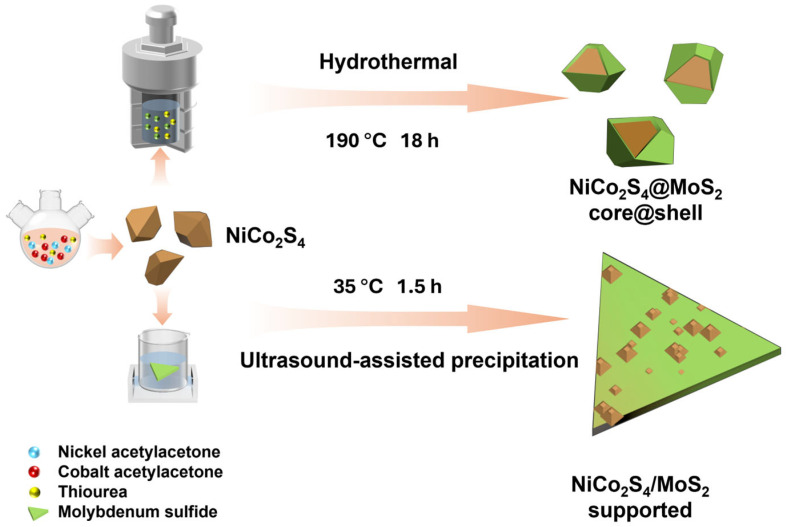
Schematic illustration for the synthesis procedures of NiCo_2_S_4_ nanoparticles, NiCo_2_S_4_@MoS_2_ core@shell heterostructure, and NiCo_2_S_4_/MoS_2_ supported heterostructure.

Figure 1 illustrates the detailed synthesis process of the NiCo_2_S_4_–MoS_2_ heterostructure. Initially, bimetallic sulfide NiCo_2_S_4_ nanoparticles were synthesized in a three-necked flask via a solvothermal method, with precise control over precursor concentration, temperature, and reaction time. Previous studies have demonstrated that bimetallic sulfides exhibit higher redox reversibility and enhanced electron/mass transport efficiency than their monometallic counterparts, thus providing a highly active foundation for the subsequent heterostructure [47,48]. Subsequently, a few layers of MoS_2_ were grown on the surface of the as-prepared NiCo_2_S_4_ nanoparticles through a hydrothermal method combined with a seed growth strategy, resulting in a stable core@shell heterostructure, denoted as NiCo_2_S_4_@MoS_2_. This architecture preserves the high electrical conductivity of the NiCo_2_S_4_ core to facilitate efficient charge collection and delivery to the interface, while the sulfur vacancy-rich MoS_2_ shell provides abundant active sites and enhanced intrinsic conductivity. The strong interfacial synergy, promoted by the heterojunction coupling and vacancy engineering, significantly optimizes the charge transfer kinetics across the interface and within the composite, collectively boosting the electrocatalytic performance. To verify this interfacial synergy, a comparative sample was prepared by employing an ultrasound-assisted method to load as-prepared NiCo_2_S_4_ nanoparticles onto commercial few-layer MoS_2_ nanosheets, yielding a supported heterostructure, denoted as NiCo_2_S_4_/MoS_2_. Because this sample lacks the unified layer-by-layer interface of the core@shell structure, its performance in electron transfer resistance and active site utilization provides a clear contrast. As shown in Table S1, we also analyzed the metal element contents of the three samples using ICP-OES. In the pristine NiCo_2_S_4_ sample, the mass ratio of Ni to Co is 16.5:31.6. Considering the relative atomic masses of Ni and Co, their atomic ratio is approximately 1:2, which further confirms the successful synthesis of the NiCo_2_S_4_ nanomaterial. In the NiCo_2_S_4_@MoS_2_ core@shell structure sample, the mass ratio of Mo to Ni and Co is comparable to that in the NiCo_2_S_4_/MoS_2_ supported heterostructure. This ensures that the influence of the MoS_2_ to NiCo_2_S_4_ mass ratio can be excluded when comparing their electrocatalytic performance.

To analyze the crystal phases of the three products, XRD measurements were performed initially. As depicted in Figure S1, the diffraction peaks align well with the reference PDF data for cubic-phase NiCo_2_S_4_ (JCPDS No. 20-0782) and 2H-phase MoS_2_ (JCPDS No. 37-1492). The supported NiCo_2_S_4_/MoS_2_ sample exhibits distinct diffraction peaks for MoS_2_, indicating its high crystallinity. In addition, three weaker diffraction peaks at 38.3°, 55.1°, and 66.4° correspond to the (400), (440), and (533) planes of NiCo_2_S_4_, respectively. For the NiCo_2_S_4_@MoS_2_ core@shell heterostructure, the face-centered cubic NiCo_2_S_4_ peaks dominate the XRD pattern. The presence of the MoS_2_ (002) peak positioned at 14.3° indicates that MoS_2_ effectively covers the NiCo_2_S_4_ surface, while the diminished intensity of other MoS_2_ peaks is probably due to the ultrathin, defect-rich characteristics of the MoS_2_ shell.

The presence of MoS_2_ was further examined using surface-sensitive Raman spectroscopy. Figure S2 illustrates that the Raman spectrum of the NiCo_2_S_4_/MoS_2_ supported heterostructure and the pristine commercial MoS_2_ nanosheets display characteristic peaks at 376 cm^−1^ and 402 cm^−1^, which correspond to the $E_{2g}^{1}$ (in-plane Mo–S bond vibration) and *A*_1*g*_ (out-of-plane vibration) modes of MoS_2_, respectively [49,50]. In the NiCo_2_S_4_@MoS_2_ core@shell heterostructure, Raman peaks in the 360  cm^−1^ to 405  cm^−1^ region are also observed, where the characteristic MoS_2_ peaks overlap and broaden. This behavior can be ascribed to the ultrathin, few-layer MoS_2_ coating on the NiCo_2_S_4_ surface, thus the $E_{2g}^{1}$ mode undergoes a blueshift while the *A*_1*g*_ mode redshifts, narrowing the separation between them, and the high concentration of S_V_s disrupts the lattice periodicity, causing disorder-induced broadening of the Raman peaks [51,52,53].

**Figure 2:**
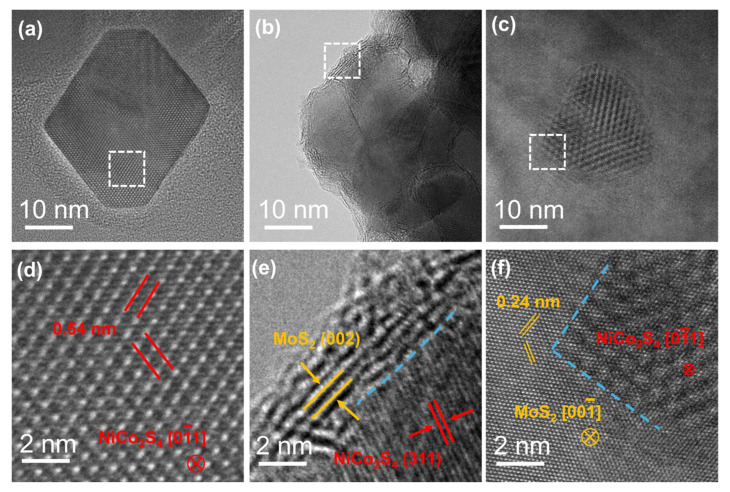
(**a**–**c**) Representative TEM images of a NiCo_2_S_4_ nanoparticle, NiCo_2_S_4_@MoS_2_ core@shell, and NiCo_2_S_4_/MoS_2_ supported heterostructures, respectively; (**d**–**f**) the HRTEM images of the selected regions marked by the white dashed frames in (**a**–**c**), where the blue dashed lines indicate the NiCo_2_S_4_–MoS_2_ hetero-interfaces.

TEM characterization was performed to further explore the morphological and atomic structural features of the samples. The low-magnification TEM image (Figure 2a and Figure S3) demonstrates that the NiCo_2_S_4_ nanoparticles possess a truncated octahedral morphology. The high-resolution TEM (HRTEM) image (Figure 2d) reveals continuous lattice fringes, indicating the high crystallinity of pristine NiCo_2_S_4_ nanoparticles. As shown in the low-magnification TEM image (Figure 2c), NiCo_2_S_4_ nanoparticles are successfully loaded onto the surface of commercial MoS_2_ nanosheets in the NiCo_2_S_4_/MoS_2_ sample. The structural integrity of MoS_2_ is preserved following the incorporation of NiCo_2_S_4_. The HRTEM image of the supported sample (Figure 2f) reveals distinct lattice fringes corresponding to the (011(−)) plane of NiCo_2_S_4_ (measured at 0.28 nm) and the (100) plane of MoS_2_ (measured at 0.24 nm). A region of reduced image contrast is observed at the interface, indicating the formation of a mixed region resulting from interactions between MoS_2_ and NiCo_2_S_4_. Figure 2b,e present the low- and high-magnification TEM images of the NiCo_2_S_4_@MoS_2_ core@shell heterostructure. Four to five layers of MoS_2_ are observed on the surface of the NiCo_2_S_4_ nanoparticles, exhibiting an interlayer spacing of 0.602 nm, which corresponds to the (002) plane of the 2H-phase MoS_2_. Compared with commercial MoS_2_, the lattice fringes of surface MoS2 are discontinuous, displaying significant distortions and dislocations, suggesting a high density of defects and a loose, porous structure. Previous studies have indicated that defects and dislocations can function as effective active sites for catalytic reactions. The loose and porous surface structure also facilitates efficient mass and electron transport during the reaction process, significantly enhancing electrocatalytic activity.

**Figure 3:**
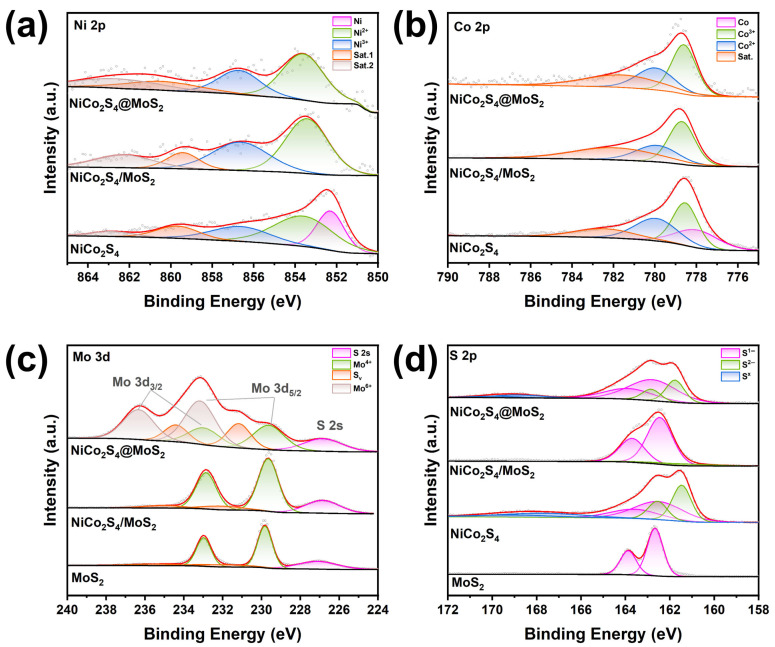
High-resolution XPS spectra of (**a**) Ni 2p, (**b**) Co 2p, (**c**) Mo 3d, and (**d**) S 2p in NiCo_2_S_4_@MoS_2_ core@shell, NiCo_2_S_4_/MoS_2_ supported heterostructures, pristine NiCo_2_S_4_ nanoparticles, and MoS_2_ nanosheets.

To understand the interfacial interactions within the heterostructures, the chemical states and electronic structure variations in the catalysts were examined by XPS. Figure S4 presents the survey spectra of the three samples, indicating the existence of Ni, Co, S, C, and O elements in the NiCo_2_S_4_ sample, while the two heterostructure samples also exhibit signals corresponding to Mo. The high-resolution spectra of Ni 2p and Co 2p for the three samples are illustrated in Figure 3a,b. In the Ni 2p spectrum of the NiCo_2_S_4_ sample, the peak at 852.3 eV corresponds to metallic Ni, while the peaks at 853.6 eV and 856.6 eV are attributed to Ni^2+^ and Ni^3+^ in the Ni 2p_3/2_ region, respectively. The satellite peaks at 859.7 eV and 862.9 eV indicate the slight oxidation of Ni species. In the Co 2p spectrum of the NiCo_2_S_4_ sample, the peak at 778.0 eV corresponds to metallic Co, while the binding energies of 778.5 eV and 780.0 eV in the Co 2p_3/2_ spectrum are characteristic of Co^3+^ and Co^2+^, respectively. The satellite peak at 782.4 eV also suggests the minor oxidation of Co species [54]. The comparative analysis of the spectra for Ni and Co elements in the three samples reveals that the peaks for Ni and Co in NiCo_2_S_4_@MoS_2_ and NiCo_2_S_4_/MoS_2_ all shift towards a higher binding energy. According to the Ni 2p deconvolution (Figure 3a), metallic Ni (Ni^0^) is transformed into Ni^2+^ and Ni^3+^ as a result of heterostructure formation, with a notable increase in the proportion of Ni^2+^ in the core@shell structure. A higher ratio of oxidized species (Co^3+^ and Co^2+^) is also observed in the heterostructures for Co 2p. The changes in the valence states of Ni and Co, along with the shifts in binding energy, indicate a reconstruction of interfacial electronic structures in the heterostructures. This phenomenon is primarily driven by electron transfer effects arising from electronegativity differences between the metals involved.

In the S 2p spectra (Figure 3d), the NiCo_2_S_4_ sample exhibits peaks at 161.5 eV and 162.6 eV, which are associated with S^2−^ species. The peaks located at 162.5 eV and 163.6 eV are characteristic of S^1−^ species. In the NiCo_2_S_4_/MoS_2_ supported heterostructure, the peaks at 162.5 eV and 163.7 eV in the XPS spectra are attributed to S^1−^, with the majority of these signals associated with MoS_2_, as verified by the spectral characteristics observed in commercial MoS_2_. In the NiCo_2_S_4_@MoS_2_ core@shell heterostructure, the S 2p spectra reveal an increased relative content of S^1−^ species in comparison to the NiCo_2_S_4_ sample, suggesting modifications in the sulfur environment resulting from the construction of the core@shell heterostructure. As shown in Figure 3c, the Mo 3d spectrum of commercial MoS_2_ displays characteristic peaks at 229.8 eV, which correspond to Mo^4+^ in Mo 3d_5/2_. The supported heterostructure also exhibits this oxidation state, suggesting the retention of Mo^4+^ species. The Mo 3d spectrum of NiCo_2_S_4_@MoS_2_ core@shell heterostructure reveals peaks at 229.6 eV and 233.2 eV, corresponding to Mo^4+^ and Mo^6+^ components, respectively. Interestingly, extra peaks were observed at 231.2 eV and 234.4 eV, which correspond to Mo species related to S_V_. The area ratio of the S_V_-related Mo peaks is approximately 25.3%. This phenomenon can be mainly explained by the reduced binding energy of sulfur atoms, which allows for their easier detachment during the synthesis process [55,56]. The higher proportion of Mo atoms at elevated oxidation states in NiCo_2_S_4_@MoS_2_, relative to commercial MoS_2_, indicates that the creation of sulfur vacancies at the interface facilitates substantial electron transfer. This conclusion is also supported by the S 2p spectrum, which reveals a greater density of sulfur vacancies, emphasizing the significance of interfacial electronic interactions in influencing the Mo oxidation states. All these XPS results confirm that the interaction between NiCo_2_S_4_ and MoS_2_ affects the binding energies of adsorbates, thereby controlling the catalytic performance of the heterostructure

**Figure 4:**
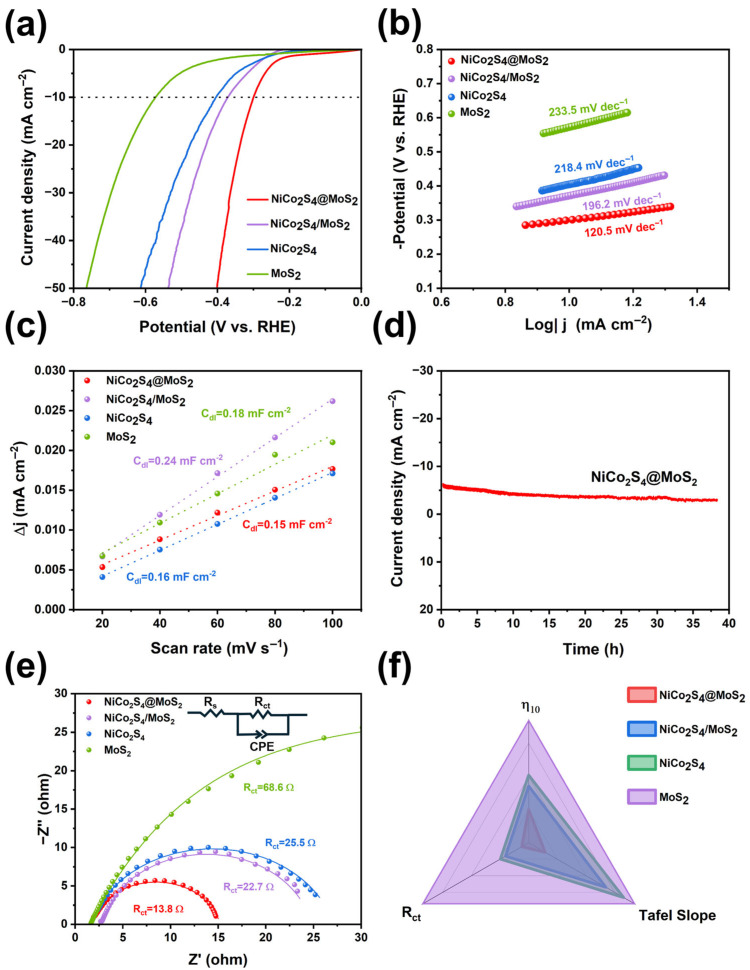
(**a**) LSV curves, (**b**) Tafel plots, and (**c**) measured C_dl_ values for NiCo_2_S_4_@MoS_2_ core@shell heterostructure, NiCo_2_S_4_/MoS_2_ supported heterostructure, NiCo_2_S_4_ nanoparticles, and MoS_2_ nanosheets in 0.5 M H_2_SO_4_ solution; (**d**) chronopotentiometric curve of NiCo_2_S_4_@MoS_2_ core@shell heterostructure; (**e**) EIS plots; (**f**) radar plots of four catalysts comparing their *η*_10_, Tafel slope, and *R*_ct_.

To evaluate the structure–property correlation of the synthesized heterostructures, HER performance tests were conducted in 0.5 mol/L H_2_SO_4_ using a three-electrode configuration. The catalytic activities of pristine NiCo_2_S_4_ and MoS_2_ were also characterized for comparison purposes. All initial LSV data were corrected with 90% *iR* compensation to exclude the influence of ohmic resistance on the intrinsic catalysis of the materials. As shown in Figure 4a, the NiCo_2_S_4_@MoS_2_ core@shell heterostructure delivers enhanced HER performance, requiring an overpotential of 300 mV to achieve a current density of 10 mA cm^−2^ (*η*_10_ = 300 mV). This catalytic performance is superior to that of the supported NiCo_2_S_4_/MoS_2_ heterostructure (*η*_10_ = 371 mV), pristine NiCo_2_S_4_ nanoparticles (*η*_10_ = 404 mV), and commercial MoS_2_ nanosheets (*η*_10_ = 573 mV). As previously discussed, the 70 mV difference in overpotential between the NiCo_2_S_4_@MoS_2_ and NiCo_2_S_4_/MoS_2_ samples can be ascribed to the elevated density of defects located at the surface and interface regions within the core@shell structure, along with the richer valence states of S and Mo atoms, resulting in a greater availability of catalytic active sites. The comparison between NiCo_2_S_4_/MoS_2_ and pristine MoS_2_ samples demonstrates that the NiCo_2_S_4_ nanoparticles can partially activate the basal plane activity of MoS_2_, leading to an approximate 30 mV improvement in overpotential.

A reduced Tafel slope typically indicates faster HER kinetics and a decreased overpotential for the electrocatalyst at a specific current density. As shown in Figure 4b, according to the LSV-derived Tafel slopes, the NiCo_2_S_4_@MoS_2_ core@shell structure demonstrates superior HER kinetics, with a Tafel slope of 120.5 mV dec^−1^, which is obviously smaller than those of the NiCo_2_S_4_/MoS_2_ supported heterostructure (196.2 mV dec^−1^) and pristine NiCo_2_S_4_ (218.4 mV dec^−1^), and considerably lower than that of the MoS_2_ nanosheets (233.5 mV dec^−1^). The values of the Tafel slope indicate that the HER catalytic mechanism of NiCo_2_S_4_@MoS_2_ predominantly adheres to the Volmer–Heyrovsky mechanism, where the initial Volmer step, involving the electrochemical adsorption of protons onto active sites, is probably the rate-limiting step. The numerous defects and sulfur vacancies at the surface and interface in the NiCo_2_S_4_@MoS_2_ core@shell heterostructure significantly reduce the energy barrier for proton adsorption. Meanwhile, the improved interfacial electron transfer promotes the subsequent electrochemical desorption of hydrogen (the Heyrovsky step).

The electrochemically active surface areas (ECSA) of the catalysts were evaluated using CV curves (Figure S5). The double-layer capacitance (*C*_dl_), indicative of the effective surface area, was derived by taking half the difference between the anodic and cathodic current densities at 0.65 V (vs. RHE) and normalizing it by the scan rate. As shown in Figure 4c, compared with NiCo_2_S_4_ nanoparticles and MoS_2_ nanosheets, the NiCo_2_S_4_/MoS_2_ supported heterostructure exhibits a higher *C*_dl_ value (0.24 mF cm^−2^), indicating that the in-plane activity of MoS_2_ is enhanced due to the loading of NiCo_2_S_4_ nanoparticles. Although the NiCo_2_S_4_@MoS_2_ core@shell heterostructure shows a smaller electrochemical active surface area than the NiCo_2_S_4_/MoS_2_ supported heterostructure, it demonstrates superior HER performance. This indicates that the active sites exhibit a higher reaction efficiency, primarily due to the strong interfacial interaction between the two components, which greatly boosts their intrinsic catalytic activity.

The *i-t* curve demonstrates that the current density remains stable over 38 h without noticeable decay (Figure 4d). This indicates that the NiCo_2_S_4_@MoS_2_ sample exhibits excellent stability, attributed to its heterostructured core@shell configuration, which effectively prevents the peeling and dissolution of active materials during the catalytic process.

The EIS curves (Figure 4e) were produced to infer the interfacial reaction and electron transfer kinetics. Among all samples, the MoS_2_ nanosheets possess the highest charge transfer resistance (*R*_ct_ = 68.6 Ω). Compared with the NiCo_2_S_4_/MoS_2_ supported heterostructure (*R*_ct_ = 22.7 Ω) and NiCo_2_S_4_ nanoparticles (*R*_ct_ = 25.5 Ω), the charge transfer resistance of the NiCo_2_S_4_@MoS_2_ core@shell heterostructure is significantly reduced to 13.8 Ω. This improvement originates from the enriched valence states of the elements, which greatly enhance the intrinsic conductivity of the NiCo_2_S_4_-based sample. As a result, it ensures rapid electron transport across the interface and optimizes the interfacial kinetics in acidic media.

**Figure 5:**
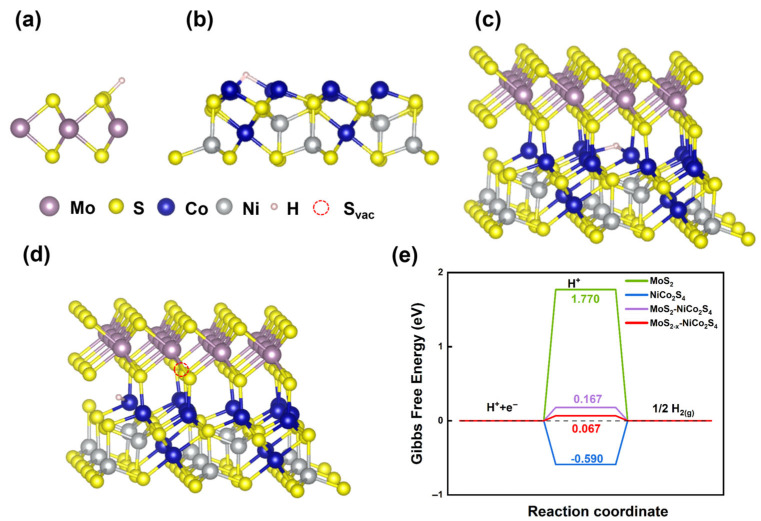
Optimized structural representations for hydrogen adsorption at (**a**) MoS_2_, (**b**) NiCo_2_S_4_, (**c**) NiCo_2_S_4_–MoS_2_ interfacial structure, and (**d**) NiCo_2_S_4_–MoS_2−*x*_ interfacial structure with sulfur vacancy; (**e**) calculated free energy of hydrogen adsorption at the above positions.

The above results indicate that constructing heterostructures and utilizing the interfacial interaction between NiCo_2_S_4_ and MoS_2_ can effectively enhance HER catalytic activity. Furthermore, in the core@shell heterostructure, the catalytic performance is the best due to more extensive interfacial contact and a higher density of surface defects. To further investigate the origin of the HER catalytic activity of NiCo_2_S_4_@MoS_2_, DFT calculations were conducted. Based on the Sabatier principle, an ideal HER catalyst should exhibit a moderate hydrogen adsorption free energy (∆*G*_H*_), neither too strong nor too weak, to balance reactant activation and product desorption [46]. As shown in Figure 5, the DFT calculations reveal that the pristine 2H-MoS_2_ basal plane binds hydrogen very weakly (∆*G*_H*_ = +1.770 eV), reflecting its inertness toward HER. In contrast, NiCo_2_S_4_ displays overly strong hydrogen binding (∆*G*_H*_ = –0.590 eV), which impedes the desorption step of HER. Forming a heterojunction between NiCo_2_S_4_ and MoS_2_ induces an interfacial charge redistribution that moderates ∆*G*_H*_ to −0.167 eV, which is much closer to the thermoneutral optimum, thereby optimizing the adsorption/desorption equilibrium and enhancing HER activity. The further introduction of sulfur vacancies exposes under-coordinated Mo sites, fine tuning ∆*G*_H*_ to −0.067 eV and creating abundant active edge centers that lower the hydrogen adsorption barrier, thereby maximizing the HER activity of the NiCo_2_S_4_@MoS_2_ core@shell heterostructure.

## 4. Conclusions

In this work, we engineered a NiCo_2_S_4_@MoS_2_ core@shell heterostructure and investigated its electrocatalytic HER performance. By directly comparing the core@shell NiCo_2_S_4_@MoS_2_ with a supported NiCo_2_S_4_/MoS_2_ heterostructure and pristine NiCo_2_S_4_ and MoS_2_, we showed that interfacial charge redistribution and lattice strain critically tune catalytic activity. Crucially, the creation of sulfur vacancies further boosts the density of active sites, dramatically accelerating HER kinetics. The NiCo_2_S_4_@MoS_2_ core@shell electrocatalyst delivers an optimized HER performance with a low overpotential (300 mV at a current density of 10 mA cm^−2^), a small Tafel slope (120.5 mV dec^−1^), and a higher reaction efficiency of active sites, attributed to its optimized charge transfer and enhanced sulfur vacancy concentration. DFT calculations corroborate these findings by showing how interfacial engineering and defect creation synergistically lower the hydrogen adsorption free energy. Further precise control over defect populations and a scalable synthesis of such heterostructures will be essential for advancing their practical applications in sustainable energy conversion.

## Data Availability

The data that support the findings of this study are available from the corresponding author upon reasonable request.

## Supplementary Materials

The following supporting information can be downloaded at: https://www.mdpi.com/article/10.3390/nano15141061/s1, Table S1. ICP-OES results of NiCo_2_S_4_@MoS_2_ core@shell heterostructure, NiCo_2_S_4_/MoS_2_ supported heterostructure, and NiCo_2_S_4_ nanoparticles. Figure S1. XRD patterns of NiCo_2_S_4_@MoS_2_ core@shell heterostructure, NiCo_2_S_4_/MoS_2_ supported heterostructure, and NiCo_2_S_4_ nanoparticles. Figure S2. Raman spectra of NiCo_2_S_4_@MoS_2_ core@shell heterostructure, NiCo_2_S_4_/MoS_2_ supported heterostructure, and MoS_2_ nanosheets. Figure S3. Low-magnification TEM image of NiCo_2_S_4_ nanoparticles. Figure S4. Full-scan XPS spectra of NiCo_2_S_4_@MoS_2_ core@shell heterostructure, NiCo_2_S_4_/MoS_2_ supported heterostructure, and NiCo_2_S_4_ nanoparticles. Figure S5. CV curves obtained with different scan rates of (a) NiCo_2_S_4_@MoS_2_ core@shell heterostructure, (b) NiCo_2_S_4_/MoS_2_ supported heterostructure, (c) NiCo_2_S_4_ nanoparticles, and (d) MoS_2_ nanosheets. Table S2. Equivalent circuit simulation parameters of NiCo_2_S_4_@MoS_2_ core@shell heterostructure, NiCo_2_S_4_/MoS_2_ supported heterostructure, NiCo_2_S_4_ nanoparticles, and MoS_2_ nanosheets.
