# Supplementary material for: Constructing Sulfur Vacancy-Rich NiCo2S4@MoS2 Core@shell Heterostructure via Interface Engineering for Enhanced HER Electrocatalysis
Source: Nanomaterials (Basel). 2025 Jul 9;15(14):1061. doi: 10.3390/nano15141061 (PMC12299294; doi:10.3390/nano15141061)
Supplement: Supplementary file 1 [file nanomaterials-15-01061-s001.zip › nanomaterials-3681921-supplementary.pdf]

Supporting Information

Constructing Sulfur Vacancy-Rich $NiCo_2S_4$@MoS_2 Core@shell Heterostructure via Interface Engineering for Enhanced HER Electrocatalysis

Ziteng Song, Yuan Liu, Peng Yin, Jie Dai, Yingying Xu, Rongming Wang * and Sibin Duan *

Beijing Key Laboratory for Magneto-Photoelectrical Composite and Interface Science, The State Key Laboratory for Advanced Metals and Materials, School of Mathematics and Physics, University of Science and Technology Beijing, Beijing 100083 (China)

* Correspondence: rmwang@ustb.edu.cn (R.W.); sibinduan@ustb.edu.cn (S.D)

Table S1. The ICP-OES results of $NiCo_2S_4@MoS_2$ core@shell heterostructure, $NiCo_2S_4/MoS_2$ supported heterostructure, and $NiCo_2S_4$ nanoparticles.

ICP	$NiCo_2S_4@MoS_2$	$NiCo_2S_4/MoS_2$	$NiCo_2S_4$
**Ni (wt.%)**	11.62	12.41	16.50
**Co (wt.%)**	22.38	25.24	31.61
**Mo (wt.%)**	17.36	16.58	/

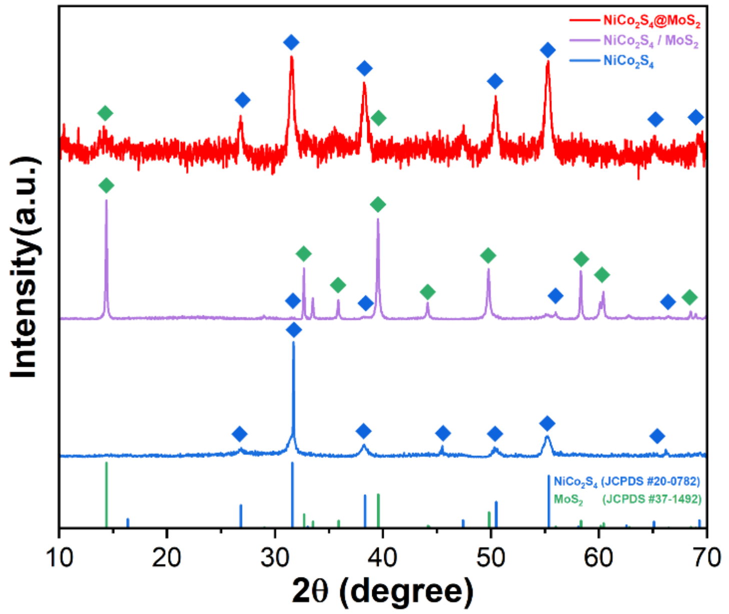

Figure S1. The XRD patterns of $NiCo_2S_4$@MoS_2 core@shell heterostructure, $NiCo_2S_4/MoS_2$ supported heterostructure, and $NiCo_2S_4$ nanoparticles.

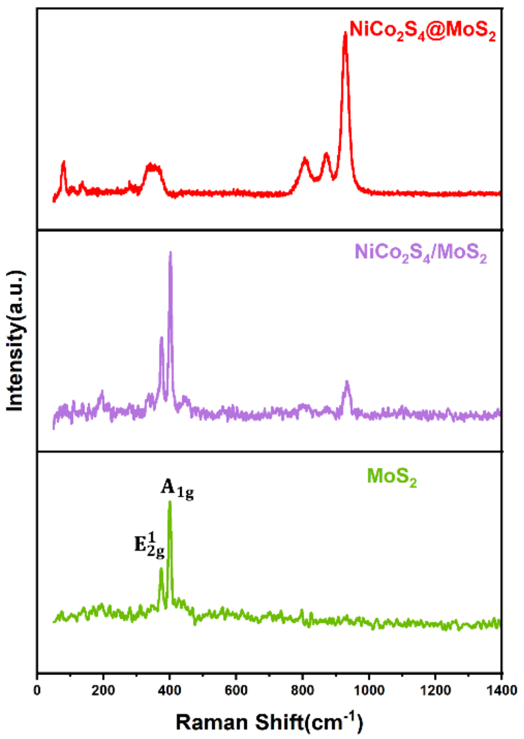

Figure S2. The Raman spectra of $NiCo_2S_4@MoS_2$ core@shell heterostructure, $NiCo_2S_4/MoS_2$ supported heterostructure, and MoS_2 nanosheets.

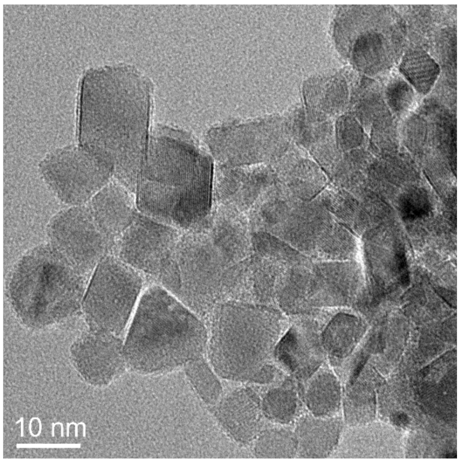

Figure S3. The low-magnification TEM image of $NiCo_2S_4$ nanoparticles.

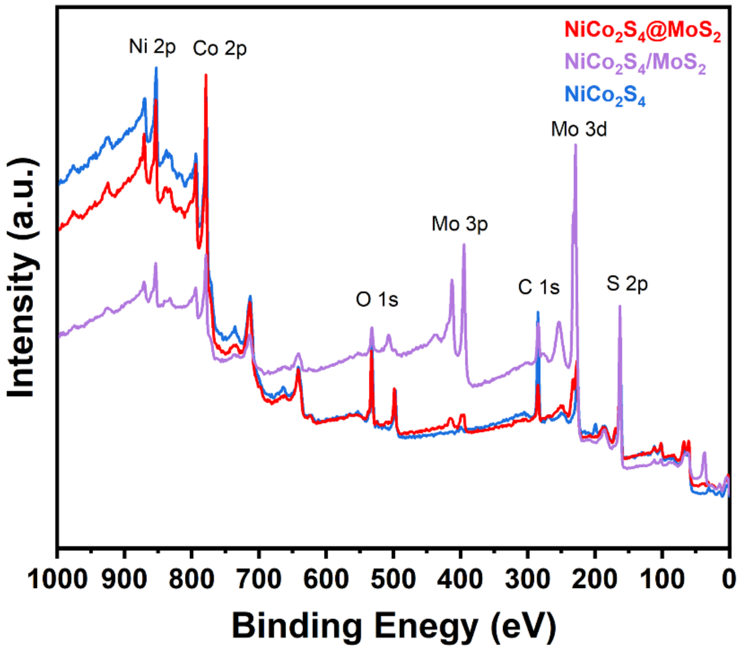

Figure S4. Full-scan XPS spectra of $NiCo_2S_4@MoS_2$ core@shell heterostructure, $NiCo_2S_4/MoS_2$ supported heterostructure, and $NiCo_2S_4$ nanoparticles.

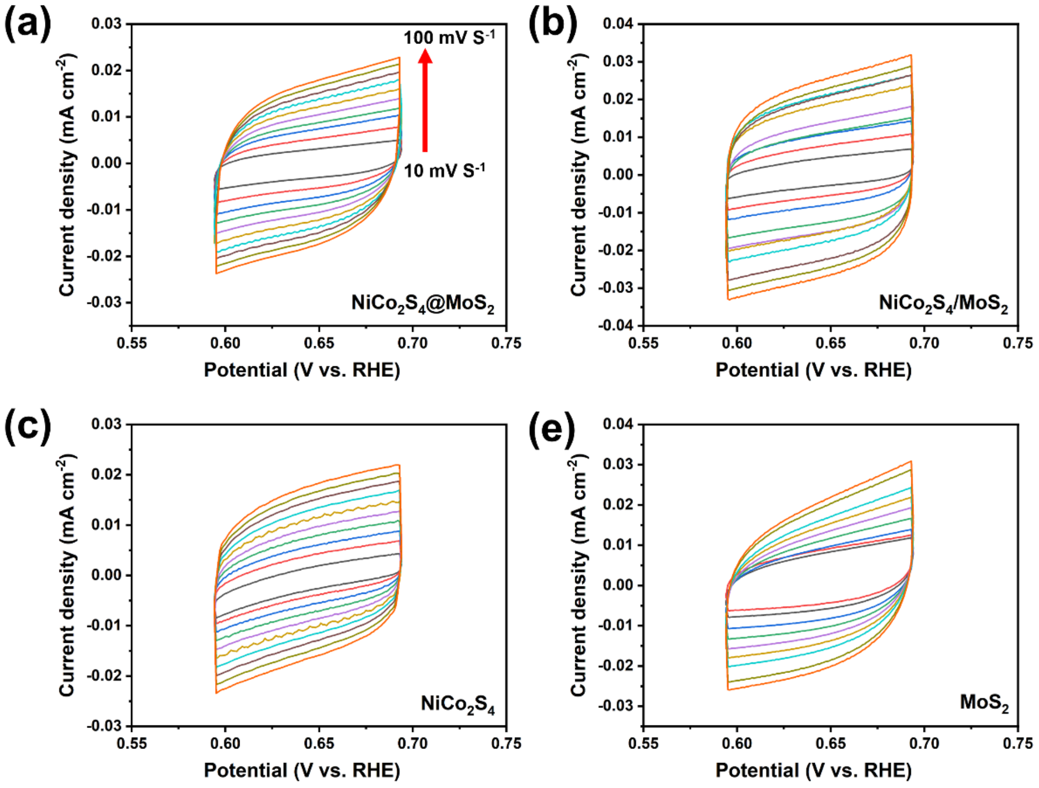

Figure S5. CV curves obtained with different scan rates of (a) $NiCo_2S_4$@MoS_2 core@shell heterostructure, (b) $NiCo_2S_4/MoS_2$ supported heterostructure, (c) $NiCo_2S_4$ nanoparticles, and (d) MoS_2 nanosheets.

Table S2. Equivalent circuit simulation parameters of $NiCo_2S_4$@MoS_2 core@shell heterostructure, $NiCo_2S_4/MoS_2$ supported heterostructure, $NiCo_2S_4$ nanoparticles and MoS_2 nanosheets

Sample	Rs (Ω)	Rct (Ω)	CPE: Y0 (S·sn)
**$NiCo_2S_4$@MoS_2**	**1.7**	**13.8**	**$1.9*10^{-4}$**
**$NiCo_2S_4/MoS_2$**	**2.8**	**22.7**	**$2.8*10^{-4}$**
**$NiCo_2S_4$**	**1.6**	**25.5**	**$2.1*10^{-4}$**
**MoS_2**	**1.7**	**68.6**	**$2.9*10^{-4}$**
